# Fetal and maternal factors are associated with mortality due to circulatory system disorders in children

**DOI:** 10.11606/S1518-8787.2019053000793

**Published:** 2019-03-26

**Authors:** Thais Rocha Salim, Gabriel Porto Soares, Carlos Henrique Klein, Gláucia Maria Moraes Oliveira

**Affiliations:** I Universidade Federal do Rio de Janeiro. Faculdade de Medicina. Instituto do Coração Edson Saad. Rio de Janeiro, RJ, Brasil; II Universidade de Vassouras. Curso de Medicina. Vassouras, RJ, Brasil; III Fundação Oswaldo Cruz. Escola Nacional de Saúde Pública. Rio de Janeiro, RJ, Brasil

**Keywords:** Child, Adolescent, Cardiovascular Diseases, mortality, Vascular Malformations, mortality, Heart Defects, Congenital, mortality, Risk factors, Infant, Premature, Asphyxia Neonatorum, Maternal Health

## Abstract

**OBJECTIVE:**

To analyze the association of characteristics recorded at the time of birth, including weight, occurrence of asphyxia, gestation duration, maternal age and education level, with death from diseases or malformations of the circulatory system in children under 18 years of age.

**METHODS:**

The Brazilian Information System on Live Births and Information System on Mortality databases were linked and evaluated following a longitudinal cohort analysis strategy. The following independent variables were evaluated: characteristics recorded at the time of birth, including weight, occurrence of asphyxia, gestation duration, maternal age and education level. Dependent variables were death from diseases or malformations of the circulatory system in children under 18 years of age. Crude relative risks were estimated and relative risks were adjusted for the variables.

**RESULTS:**

6,380 deaths were linked to 4,282,260 birth records, yielding 5,062 pairs considered as true. Low birth weight (RR = 2.26), asphyxia at 1 (RR = 1.72) and 5 minutes (RR = 1.51), prematurity (RR = 1.50), maternal age ≥ 40 years (RR = 2.06), and low maternal education level (RR = 1.45) increased the probability of death caused by circulatory system diseases. In the association with death by malformations of the circulatory system, the predictive variables showed the same association profile, but with greater intensity.

**CONCLUSIONS:**

Fetal and maternal factors are associated with increased mortality due to diseases and malformations of the circulatory system. Measures to control these factors and improve access to their diagnosis and treatment would contribute to reducing the number of deaths caused by diseases and malformations of the circulatory system. However, the identification of environmental influences during gestation and birth on the risk of death should be carefully considered due to being influenced by genetic factors.

## INTRODUCTION

In recent decades, diseases of the circulatory system (DCS) in adults have been associated with high morbidity and mortality in Brazil and worldwide ^1^ ^,^ ^2^ . Research on the risk factors associated with DCS has been continuous, since the knowledge about and acting on these factors may decrease morbidity and mortality associated with DCS. However, traditional risk factors such as obesity, hypertension, dyslipidemia, smoking, sedentary lifestyle, diabetes, and insulin resistance require a prolonged exposure duration to manifest atherosclerotic disease and death; therefore, their effects may not be detectable in individuals under the age of 18 years^3–8^. At this early stage of life, other factors contributing to the occurrence of deaths due to DCS must occur, especially conditions present at birth, such as the child’s weight, the presence of asphyxia, gestation duration, and maternal factors ^9^ ^,^ ^10^ .

David Barker’s hypothesis about a fetal origin for cardiovascular diseases suggests that environmental exposures to intrauterine risk factors affect fetal development and increase the risk of DCS, diabetes, and metabolic syndrome in adulthood ^9^ . Early studies in this field have investigated whether weight and head circumference at birth could predict the occurrence of DCS in adulthood, and whether low birth weight and the parents’ social stratum could predict an increased mortality from these diseases ^10^ ^,^ ^11^ . Low birth weight has been associated with high cholesterol concentrations, increased risk of hyperlipidemia and type 2 diabetes, and death due to DCS in adulthood^10–14^. However, there are no studies on the association of birth conditions with death by DCS in children and adolescents. On the other hand, regarding deaths due to malformations of the circulatory system (MCS), studies reveal their association with birth conditions such as prematurity and low birth weight ^15^ ^,^ ^16^ .

The objective of this study was to verify the association between characteristics present at birth, such as weight, asphyxia, gestation duration, and maternal age and education level with death due to DCS and MCS in children under 18 years of age, born and deceased between 1996 and 2014 in the state of Rio de Janeiro, Brazil.

## METHODS

This study was conducted using preexisting official databases, which were linked and evaluated using a longitudinal cohort analysis strategy. The databases analyzed were the Information System on Live Births ( *Sistema de Informações Sobre Nascidos Vivos* – SINASC, http://sistemas.saude.rj.gov.br/tabnet/deftohtm.exe?sinasc/nascido.def) of the state of Rio de Janeiro from 1996 to 2014, which collects information from birth certificates (BC), and the Mortality Information System ( *Sistema de Informações de Mortalidade* – SIM, http://sistemas.saude.rj.gov.br/tabnet/deftohtm.exe?sim/obito.def), which collects information from death certificates (DC) also from 1996 to 2014 and restricted to deaths of individuals under 18 years of age. Both SINASC and SIM are available as annual databases provided by the State Health Department of Rio de Janeiro.

From the SINASC, data on birth weight, Apgar score at 1 and 5 minutes, gestation duration, and maternal age and education level were collected; from the SIM database, the deaths whose underlying cause were DCS (coded in Chapter IX of the International Classification of Diseases, ICD-10) ^17^ and MCS (from Q20 to Q28) were selected. From the DCS group, the following specific causes were used: rheumatic fever (I00-09); hypertensive diseases (I10-15); ischemic heart diseases (I20-25); pulmonary heart disease and diseases of pulmonary circulation (I26-28); membrane diseases (pericarditis, I30-I32, and acute and subacute endocarditis, I33); valvular diseases (I34-39); myocarditis (I40-41); cardiomyopathies (I42-43); conduction disorders (I44-49); heart failure (I50); complications of heart disease and ill-defined heart diseases (I51-52); cerebrovascular (CVD) and hemorrhagic diseases (I60-62) and other cerebrovascular diseases (I63-69); and other unspecified DCS (I70-99). Deaths from MCS were divided into the following categories: chambers and cardiac connections (Q20); cardiac septa (Q21); pulmonary and tricuspid valves (Q22); aortic and mitral valves (Q23); others and unspecified (Q24); great arteries (Q25); and other vessels (Q26-28).

Predictive variables were categorized as follows: 1) birth weight as low (less than 2,500 g), adequate (2,500 g to 3,999 g), and high (4,000 g or more); 2) Apgar score at one and five minutes of life as the occurrence of asphyxia (scores 0 to 7) and normal (from 8 to 10); 3) gestational age as preterm (less than 37 weeks), term (from 37 to 41 weeks), and post-term (more than 42 weeks); 4) maternal age as below 20 years, 20 to 29 years, 30 to 34 years, 35 to 39 years, and ≥ 40 years; and 5) maternal education level as high (12 or more years of study), medium (nine to 11 years of study from 1996 to 1999, and from eight to 11 from 1999 to 2014), elementary (1^st^ grade or from one to seven years from 1996 to 1999, and from one to seven years from 1999 to 2014), and no schooling.

Statistical analyses were performed using the computer program Stata^®^, version 12 ^18^ . To evaluate the association between the information on the predictive variables at the time of birth with deaths due to DCS and MCS, the databases were liked by two methods: deterministic and probabilistic. Only the BC of single births were selected for linkage. The deterministic method used common shared information on the death and birth databases: the live birth certificate number, a unique identifier in both bases, only used from 1999 onwards in the DC of children under one year of age. Due to this deterministic method resulting in an incomplete linkage, a complementary probabilistic method was necessary. For probabilistic linkages, the mother’s name and the child’s date of birth and sex were used. For this linkage method, the reclink routine in the Stata program was used, it generates a relationship score ranging between 0 and 1. A 1 score was obtained when association keys were identical in both databases. True birth and death pairs were considered as all those with a 1 score, regardless of the common identification variable (BC number in the DC) coinciding or not. For those in which the score was below 1 but ≥ 0.99, a manual revision was conducted, accepting as true pairs those in which the differences in the strings of the mother’s names were restricted to letters swapped, added, removed, or repeated, with no space or with an additional space between the first and last names, *da* or *de* particles, accent marks, omitted apostrophes or cedillas, incomplete names, and abbreviated surnames. Linkage was not only probabilistic but also deterministic in cases in which the BC number was the same in the BC and in the DC, regardless of the score obtained in the reclink routine. Other pairs with scores below 0.99 were not considered to be true pairs, since the differences in the name strings offered no safety for the linkage, unless the same BC number was in the BC and in the DC, or if during manual revision they presented the same criteria used for ≥ 0.99 scores.

Data were analyzed using a cohort strategy in which the outcome was death by DCS or MCS. Cohorts were formed by the categories of the predictive variables in live births from single deliveries, and the crude and adjusted relative risks (RR) of death by DCS or MCS. Confidence intervals were not estimated because the results are not estimates with sample error because they derive from census data for both deaths and births. Crude estimates were obtained using binary models (binreg routine, with an RR argument in Stata^®^), in which each one contains only one of the predictive variables. Adjusted estimates were obtained with models that included all predictive variables. The population attributable fractions for the risk of death due to DCS or MCS were also estimated in the categories of predictive variables. The deaths identified for each cohort of predictive variable category would depend on the efficiency of the linkage between the SINASC and SIM databases so estimates for the risk of death could be underestimated with linkage efficiency below 100%. However, RR would not be underestimated in the absence of differentials in linkage efficiency in the categories. To ensure that these differentials did not occur, the probabilistic linkage process was performed without knowledge of the categories of the predictive variables.

The study was performed in accordance with the current ethical principles and was approved by the Research Ethics Committee of Hospital Universitário Clementino Fraga Filho of Universidade Federal do Rio de Janeiro.

## RESULTS

**Figure 1:**
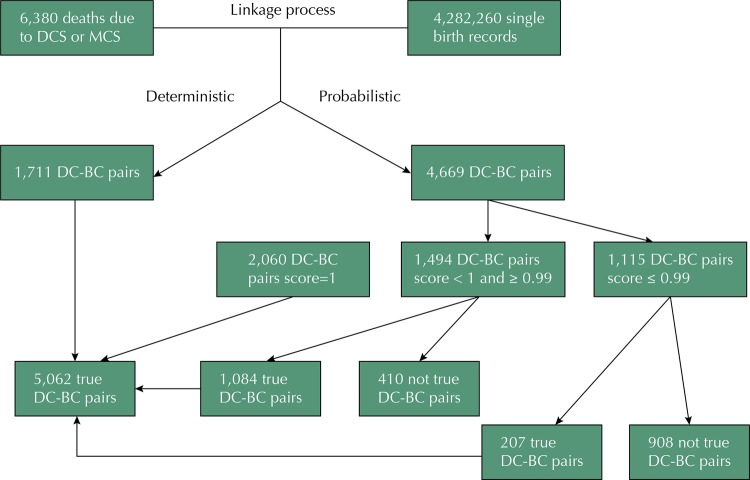
Flowchart showing the steps for linkage of the pairs death certificate (DC) and birth certificate (BC).

This study linked 6,380 deaths due to DCS or MCS to 4,282,260 single birth records, yielding 5,062 pairs considered true. Figure 1 shows the quantitative results of the linkage steps, which comprised a deterministic and a probabilistic branch. In the deterministic branch, DC-BC pairs were considered true because the identification numbers of live births recorded in the BC and in the DC coincided. Such deterministic linkage was only possible for children aged below one year. The other pairs were submitted to probabilistic linkage. Of 1,200 deaths by DCS, 858 (71.5%) were linked as true DC-BC pairs, and of the 5,180 deaths due to MCS, 4,204 (81.2%) were linked as true DC-BC pairs. Probabilistic linkage presented 71.8% general efficiency; however, when the contribution of the deterministic linkage was also considered, the overall DC-BC pairs percentage was 79.3%.

Of the deaths due to DCS successfully linked to birth records, 3.7% were from children who died before completing one month of life, 43.4% from children aged between one and 11 months, and 52.9% from children aged between one and 17 years. Of the deaths due to MCS successfully linked to birth records, 51.5% were from children who died before completing one month of life, 38.4% from children aged between one and 11 months, and 10.1% from children aged between one and 17 years.

As shown in Table , information losses on the status of the risk factors were under 5.5%; thus, the sums of the quantities of DCS and MCS pairs in all categories of each variable do not equal to the total of true pairs. In the case of Apgar at five minutes, this loss reached 5.2% for DCS deaths, and in the case of the Apgar at one and five minutes, information loss was 5.5% for MCS deaths. Information coverage on maternal age was almost 100%.

**Table:** Crude and adjusted relative risks of death due to diseases or malformations of the circulatory system relative to the population of live births according to predictive variables in children below the age of 18 years in the state of Rio de Janeiro, from 1996 to 2014.

Predictive variables	DCS (n)^a^	MCS (n)^a^	Live births (n)^a^	DCS			MCS		
	
				RR crude	FAP (%)	RR adjusted^b^	RR crude	FAP (%)	RR adjusted^b^
Birthweight									
Adequate	611	2,619	3,715,583	1	-	1	1	-	1
Low	196	1,335	342,531	3.48	17.30	2.26	5.53	27.64	2.96
High	34	146	217,069	0.95	-0.26	0.96	0.95	-0.25	0.89
Apgar 1									
Normal	510	2,046	3,294,362	1	-	1	1	-	1
Asphyxia	309	1,927	842,365	2.37	21.80	1.73	3.68	35.31	2.10
Apgar 5									
Normal	709	3,046	3,971,697	1	-	1	1	-	1
Asphyxia	104	927	166,137	3.51	9.14	1.52	7.28	20.11	2.60
Gestational age									
Term	668	2,952	3,876,594	1	-	1	1	-	1
Preterm	163	1,076	309,138	3.06	13.20	1.49	4.57	20.85	1.40
Post-term	7	47	49,435	0.82	-0.22	0.86	1.25	-0.31	1.29
Maternal age (years)									
20 to 29	400	1,957	2,231,102	1	-	1	1	-	1
< 20	203	772	842,523	1.34	8.6	1.17	1.04	1.21	0.93
30 to 34	127	677	736,367	0.96	-0.94	0.96	1.05	1.18	1.06
35 to 39	73	442	364,377	1.12	1.62	1.10	1.38	5.10	1.31
≥ 40	39	254	95,745	2.27	4.97	2.05	3.02	7.68	2.53
Maternal education level									
High	102	571	715,828	1	-	1	1	-	1
Middle	301	1,597	1,625,828	1.30	17.20	1.26	1.23	13.83	1.23
Elementary and no schooling	421	1,842	1,849,316	1.60	30.11	1.48	1.25	15.19	1.15
No information	17	97	85,377	1.40	4.06	1.22	1.42	4.32	1.12

DCS: diseases of the circulatory system (chapter IX of ICD-10); MCS: malformations of the circulatory system (Q20-28 of ICD-10); FAP: fraction attributable to population

^a^ Information losses on the status of the risk factors were under 5.5%, for this reason, pairs in all categories of each variable do not make up the total of true pairs.

^b^ Adjusted estimates were obtained with models that included all predictive variables.

The RR of death by DCS among children with low birth weight was 2.3 times higher than that observed in those with adequate birth weight after adjustment for the effect of other variables. High birth weight was not relevantly associated with death by DCS. Apgar at one or five minutes also showed a relevant association with death by DCS. The association of the gestation duration with the risk of death by DCS, both in terms of categorization level and intensity of association, showed similarities with the associations between low birth weight and death by DCS or MCS. Regarding maternal age, taking the most frequent age group (20–29 years) as a reference, the category that showed a relevantly higher risk of death was that of mothers aged 40 years or older. Regarding maternal education level, taking the upper level as a reference, both crude and adjusted RR showed a positive association between low education level and death by DCS. In the association with death by MCS, the predictive variables showed a similar association profile to that observed for those with DCS.

Table also shows the fractions attributable to the risk categories for each predictive variable in the population. An increase in years of study seemed to imply in a reduction of almost 30% in deaths by DCS. Deaths by MCS would reduce by about one-third in the absence of asphyxia at 1 minute.

**Figure 2:**
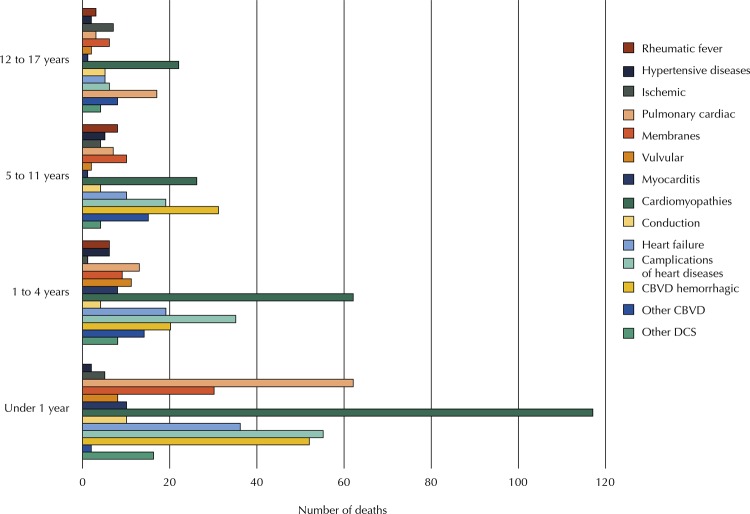
Number of deaths due to diseases of the circulatory system by age group below the age of 18 years, state of Rio de Janeiro, 1996 to 2014.

CBVD: cerebrovascular diseases; DCS: diseases of the circulatory system

Figure 1 shows the number of deaths due to specific causes in the DCS set according to the age range. In this set, the main cause of death was cardiomyopathy in all age groups, except for the 5–11 years age range, in which hemorrhagic cerebrovascular disease was found as the main cause, closely followed by cardiomyopathy. Figure 2 shows the number of deaths due to specific causes in the MCS set, in which the main causes were the unspecified ones, followed by septal and large artery malformations, except in the group below one year of age, in which these last two causes were inverted.

**Figure 3:**
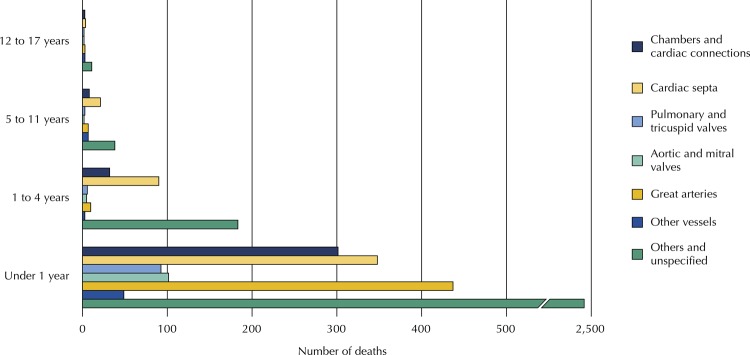
Number of deaths due to malformations of the circulatory system by age group of people under 18 years of age, state of Rio de Janeiro, 1996 to 2014.

## DISCUSSION

This study used databases linked by probabilistic and deterministic linkage to study the predictive variables of mortality associated with diseases and malformations of the circulatory system. Linking all DC with the underlying cause of DCS or MCS with their corresponding BC was impossible. The percentage of DC-BC pairs obtained (80%) was similar to percentages obtained in another study that combined the same databases ^20^ . We must stress that this study included relatively old SINASC databases (from the last decade of the 20^th^ century), a period when the registration quality was not yet consolidated. Death risk by DCS or MCS estimates would certainly be underestimated because the DC-BC linkage was incomplete. However, such underestimation does not compromise the RR estimates since no differential underestimations of risk in the categories of predictive variables were found. We also highlight that most associations correspond to deaths that occurred in the first year of life. The possibilities of a DC-BC linkage decreased as the age at death increased. The difference in the success in the DC-BC linkage observed in deaths by DCS and MCS (higher for MCS) occurred because a higher proportion of deaths by MCS occurred in the first year of life, in which the relative contribution of deterministic linkage was higher.

Fetal factors such as low birth weight, asphyxia, and prematurity, as well as maternal factors such as education level and age, are not only relevantly associated with the risk of death by DCS or MCS but are also relevant when the expressive values of population attributable fractions are considered.

A possible relationship between low birth weight and deaths by MCS is the overall impairment in intrauterine development in congenital malformations, increasing the risk for low birth weight alone or combined with preterm labor, which also increases the risk of prematurity and neonatal asphyxia ^15^ ^,^ ^16^ . Low birth weight may also be related to angiogenesis, in addition to being related to the embryology of the circulatory system by mechanisms not yet elucidated, leading to or aggravating the MCS and increasing the probability of death ^13^ ^,^ ^15^ . For deaths by DCS, several studies have shown a relationship with low birth weight, but no pathophysiological mechanism has been established in this regard^12–15,20–22^.

The Apgar score is a scoring system described in 1950 to evaluate the newborn and the need for interventions during labor ^23^ . The score is used to assess asphyxia at birth and predict adverse neonatal results ^24^ . Asphyxiation is a condition of decreased oxygen concentration in the blood, which, when persistent, leads to progressive hypoxemia, hypercapnia, and decrease in tissue oxygenation, which can cause serious damage to the central nervous system, respiratory and cardiovascular systems, as well as renal complications ^25^ . Therefore, the longer the duration of the asphyxia, the greater its association with deaths by DCS. The occurrence of hypoxia during embryonic development may result in changes leading to MCS. Thus, hypoxia in individuals with MCS may be a condition prior to birth that is perceived at birth by the Apgar score. On the other hand, cyanotic cardiac malformations may lead to an error in the Apgar score evaluation, since one of the criteria evaluated in the score is the color represented by the presence or absence of cyanosis in the newborn ^23^ .

Prematurity is associated with delivery abnormalities leading to increased inflammatory and hormonal factors, changing the organic homeostasis and acting as a risk factor for DCS and for MCS severity, contributing to death ^26^ . Prematurity may also be associated with other variables predictive of death, such as low weight and asphyxia.

Regarding maternal age, the highest risk of death by DCS and MCS was found in mothers aged 40 years or older. The increased risk of death by MCS was already perceived from 35 years of age onwards. This observation is corroborated in the literature since the risk of malformations and genetic syndromes is directly associated with advanced maternal age ^27^ ^,^ ^28^ .

Maternal education level was used as a variable representing the maternal socioeconomic level since it is the one with the largest coverage range in the SINASC database. The increased risk of death by MCS was related to a lower education level. Individuals with a lower education level have impaired access to the diagnosis and treatment of MCS ^29^ . This can be indirectly perceived by the fact that MCS deaths had no specified cause. Providing adequate life support and treatment for MCS is impossible without a specific diagnosis. The most affected age group is the one below one year of age and 1–4 years of age because MCS are often incompatible with life and highly dependent on adequate medical and hospital support, leading to early mortality.

Low maternal education level is also a risk factor for DCS, since social adversities during the prenatal period are independent risk factors for inflammation in adults, and this inflammatory condition, in turn, predisposes the infant to DCS and deaths because of it ^26^ . In this study, we verified that the risk of death by DCS during childhood and adolescence was higher in children of mothers with low education level. However, the most relevant specific causes of DCS death below 18 years are different from those observed in adults; in adults, the causes are related to atherosclerosis, whereas in children and adolescents, cardiomyopathies are the main cause of death ^28^ .

After adjustment for each predictive variable, their RR were attenuated, especially those for fetal factors. This must have occurred because birth weight, Apgar at 1 and 5 minutes, and gestation duration should be correlated so the adjusted association strength were lower than the crude ones.

The distribution of the basic causes of deaths due to DCS according to the age group at death indicated possible errors in diagnosis or coding since ischemic, valvular and hypertensive causes are unlikely in children younger than one year. Deaths in this age group are usually associated with congenital conditions and MCS. Similar to deaths from rheumatic fever and valvular causes, these deaths in children aged 1–4 years would be related to other clinical diagnoses, such as sepsis due to infectious arthritis, endocarditis, juvenile idiopathic arthritis, and MCS itself ^27^ ^,^ ^28^ . On the other hand, in deaths by MCS, those with unspecified causes were the most frequent, especially in children under one year of age. This suggests the occurrence of poor access to diagnosis during prenatal care and at birth, hindering an adequate treatment. Measures such as prenatal care and obstetric echocardiography could reduce these deaths, allowing an early diagnosis and referral of these patients to specialized care centers even before birth ^16^ .

One of the critical limitations of this study was the monitoring of births until the occurrence of death through probabilistic linkage between the SIM and SINASC databases, which does not provide an ideal certainty degree on the born-deceased pairs. If the DC registration identification number was also present in the BC the certainty degree would be better and remove the necessity of performing probabilistic linkage, thus limiting the analysis to the deterministic one. Another limitation was the use of official databases. The information is provided by the institutions performing the delivery, which do not follow a common protocol or research planning. Moreover, there was no protocol for calibration of the instruments used, such as the scales. Another limitation was the time interval of the study, only 19 years; such interval was used due to the availability of data on SINASC, which resulted in a database that privileged younger people and did not reproduce an age pyramid at any time. Therefore, it was impossible to observe what happened until 18 years of age, except for those born in 1996. Regarding MCS, this limitation was small, since most deaths occurred before five years of age. However, regarding DCS, we must recognize that, although moderately, the risk of death progressively increased, at least up to the age of 18 years. There was no significant variation in the outcomes considered in this study over the period. Furthermore, if databases were available for a longer period and had their quality assured by common protocols, other predictive variables could be evaluated, not only in children under 18 years of age but also in young adults ^30^ . The quality of filling out the DC was also not verified or assured by a common research protocol. However, death and birth certificates are the most comprehensive data sources due to being mandatory for all births and deaths.

## CONCLUSION

Low birth weight, presence of asphyxia at one and five minutes, prematurity, maternal age ≥ 40 years, and low maternal education level were associated with increased mortality by DCS and MCS under 18 years of age. Measures to control these variables would contribute to reduce deaths caused by these conditions among the population. However, the identification of environmental influences during pregnancy and birth on the risk of death by DCS or MCS should be carefully considered, as they are influenced by genetic factors.
